# Efficacy and safety of intravitreal injection of aflibercept biosimilar for treating diabetic macular edema

**DOI:** 10.3389/fmed.2025.1528104

**Published:** 2025-03-24

**Authors:** Gaixia Zhai, Chao Sun, Xia Zhang, Yuanzhen Su

**Affiliations:** Zibo Central Hospital, Zibo, China

**Keywords:** aflibercept biosimilar, diabetic macular edema, foveal avascular zone, vascular density, multifocal electroretinography

## Abstract

**Purpose:**

This study aimed to evaluate the efficacy and safety of intravitreal injection of aflibercept biosimilar in the treatment of diabetic macular edema (DME).

**Methods:**

Clinical data were collected from 33 patients (40 eyes) newly diagnosed with DME in the ophthalmology department of our hospital between February and April 2024, all of whom were treated with the aflibercept biosimilar. Patients were managed according to the 3+ Pro re nata (PRN) regimen and completed a minimum follow-up period of 6 months. The best-corrected visual acuity (BCVA) testing, optical coherence tomography, optical coherence tomography angiography, and multifocal electroretinography were performed before and after treatment. BCVA and central retinal thickness (CRT) were compared at baseline and 1-, 3-, and 6-months post-treatment. Additionally, the changes in the foveal avascular zone area, vascular density (VD) of superficial and deep retinal capillaries in the macular region, and the first positive peak amplitude density in ring 1 were analyzed 6 months post-treatment.

**Results:**

BCVA improved significantly from 0.53 ± 0.12 logMAR at baseline to 0.31 ± 0.12, 0.26 ± 0.10, and 0.26 ± 0.08 logMAR at 1-, 3-, and 6-months post-treatment, respectively, (*p* < 0.05). CRT decreased significantly from 422.4 ± 63.04 μm at baseline to 294.7 ± 47.89, 272.1 ± 47.43, and 281.0 ± 40.72 μm at 1-, 3-, and 6-months post-treatment, respectively, (*p* < 0.05). The foveal avascular zone area significantly reduced from 0.40 ± 0.08 mm^2^ at baseline to 0.35 ± 0.07 mm^2^ at 6 months post-treatment. Superficial VD increased significantly from 38.90 ± 7.88% at baseline to 41.21 ± 7.98% at 6 months post-treatment, while deep VD significantly increased from 35.67 ± 7.50% at baseline to 38.72 ± 6.90% (*p* < 0.05). The first positive peak amplitude improved significantly from 55.30 ± 9.45 to 72.90 ± 7.44 nv/deg^2^ at 6 months post-treatment (*p* < 0.05).

**Conclusion:**

Intravitreal injections of aflibercept biosimilar can significantly reduce DME, improve BCVA, enhance macular perfusion, and restore macular function.

## 1 Introduction

Diabetic macular edema (DME) is the leading cause of vision loss among patients with diabetes, affecting approximately 21 million individuals worldwide (1). The primary pathological mechanisms driving DME include increased vascular permeability and leakage, with vascular endothelial growth factor (VEGF) playing a pivotal role in its onset and progression (2). Given the profound socio-economic impact of DME, its prevention and timely, effective treatment have emerged as critical priorities in public health.

In recent years, intravitreal anti-VEGF therapy (3, 4) and dexamethasone implants have become the cornerstone of DME management (5–7). Conbercept, aflibercept, and ranibizumab are commonly used anti-VEGF agents for the treatment of DME. Compared to conbercept (Chengdu Kang Hong Biotechnology Co., Ltd.) and ranibizumab (Lucentis; Genentech, Inc., South San Francisco, CA, USA), aflibercept (Eylea; Regeneron, Inc., Tarrytown, NJ, USA) stands out due to its broader target range, stronger binding affinity, and longer duration of action. Despite its superior therapeutic efficacy, the high cost of aflibercept may limit its widespread adoption. The aflibercept biosimilar QL1207 developed by Qilu Pharmaceutical Co., Ltd., received certification from the National Medical Products Administration on 18 December 2023. This biosimilar is indicated for the treatment of neovascular age-related macular degeneration and DME. Its primary advantage over the originator drug lies in its cost-effectiveness, which can significantly alleviate the financial burden on patients and improve treatment adherence.

Currently, only a limited number of clinical studies have investigated the therapeutic effect of aflibercept biosimilars in the treatment of DME. Some studies suggest that frequent anti-VEGF therapy can delay the progression of retinal capillary occlusion, while others hold a contrasting view (8). The potential for anti-VEGF therapy to exacerbate retinal ischemia remains a subject of ongoing debate. In recent years, optical coherence tomography (OCT) angiography (OCTA) has emerged as a non-invasive diagnostic tool, enabling precise measurement of the foveal avascular zone (FAZ) and vascular density (VD) in both the superficial and deep retinal capillary networks of the macular region (9).

Multifocal electroretinography (mfERG) has gained recognition as an objective technique in the field of visual electrophysiology. This method provides a direct assessment of various retinal functions, with its central response offering valuable insights into macular functionality (10). Clinically, mfERG is widely utilized to evaluate the severity of retinal lesions and to monitor improvements following pharmacological interventions (11–13). The primary objective of this study was to evaluate the efficacy and safety of aflibercept biosimilars in the treatment of DME, utilizing OCTA and mfERG as key diagnostic modalities.

## 2 Methods

### 2.1 Study protocol

This study was conducted in accordance with the principles of the Helsinki Declaration and received approval from the Medical Ethics Committee of Zibo Central Hospital. The study was retrospective. However, all patients were thoroughly informed of the potential risks and clinical necessity of the intravitreal injection prior to the procedure. Written informed consent was obtained from each patient before the administration of the treatment.

### 2.2 Patients

Clinical data were collected from patients diagnosed with DME for the first time in the ophthalmology department of our hospital and subsequently treated with aflibercept biosimilars. The inclusion criteria were as follows: (a) a first-time diagnosis of DME with no prior ocular treatments administered, (b) age over 18 years, and (c) availability of complete clinical data. The exclusion criteria included: (a) a history of ocular trauma, surgery, and corneal diseases; (b) refractive media opacity that could interfere with fundus examination; (c) current use of contact lenses; (d) a history of ocular conditions such as glaucoma and optic neuritis; and (e) poorly controlled blood glucose or blood pressure levels.

### 2.3 Examination and treatment

All patients underwent intravitreal injections of aflibercept biosimilars (2 mg/0.05 mL). Treatment followed the 3+ Pro re nata (PRN) regimen, wherein the first three injections were delivered at 4-week intervals. Subsequent injections were determined based on clinical indicators. The criteria for reinjection were as follows: (1) a decline in BCVA of more than one line on the Snellen chart; (2) a CRT exceeding 280 μm; and (3) the presence of new, recurrent, or persistent subretinal or intraretinal fluid, as identified on any OCT scan. All patients completed a minimum follow-up period of 6 months. Comprehensive ophthalmic evaluations were conducted before and after treatment, including slit lamp biomicroscopy, tonometry, best-corrected visual acuity (BCVA) testing, OCT, OCTA (Optovue RTVue xR Avanti, Optovue Inc. USA), fundus fluorescein angiography (Spectralis, Heidelberg, Germany), and fundus examination. MfERG was performed at baseline and 6 months post-treatment. Central retinal thickness (CRT) was measured using OCT (Optovue, Inc., Fremont, CA, USA), while the FAZ area and VD of the superficial and deep retinal capillaries were assessed using OCTA. The first positive peak (P1) amplitude density (Amp-P1) in ring 1 was quantified using mfERG.

To rule out any surgical contraindications, all patients underwent preoperative assessments, including lacrimal irrigation, electrocardiogram, complete blood count, coagulation profile, biochemical tests, and pre-transfusion screening. To prevent intraocular infection, patients were instructed to administer levofloxacin eye drops (Santen, Japan) four times daily for 3 days prior to the procedure. All surgeries were performed under strict aseptic conditions. The surgical protocol included preoperative mydriasis using compound tropicamide eye drops and surface anesthesia with three applications of procaine hydrochloride eye drops. Patients were positioned supine on the operating table, and standard disinfection and draping procedures were followed. The conjunctival sac was disinfected with 50 g L-1 povidone-iodine and then rinsed with normal saline. Using a 30-G needle, aflibercept biosimilars (2 mg/0.05 mL) were injected into the vitreous cavity through the pars plana, 3.5–4 mm posterior to the temporal limbus. Following the injection, gentle pressure was applied to the puncture site using a sterile cotton swab. Postoperatively, an eye ointment containing tobramycin and dexamethasone was applied, and the affected eyes were covered with sterile gauze. Patients were prescribed tobramycin and dexamethasone eye drops to be used four times daily for 1 week after the surgery.

### 2.4 Observation parameters

The BCVA (logMAR) and CRT were evaluated at baseline and at 1-, 3-, and 6-months post-injection. The FAZ areas, VD of the retinal capillaries, and Amp-P1 values were assessed at baseline and 6 months following the injection.

### 2.5 Statistical analysis

In this study, GraphPad Prism 9 software was used for the statistical analysis, and the BCVA was converted to the logarithm of the minimum angle of resolution (logMAR). Quantitative data were expressed as mean ± standard deviation (SD). Repeated measures one-way analysis of variance was used to evaluate BCVA and CRT at baseline and at 1-, 3-, and 6- months post-treatment. The Anderson–Darling test was used to assess the normality of the data distribution. When the standard normal distribution was satisfied, the paired sample *t*-test was used to compare the VD and FAZ areas of retinal capillaries and Amp-P1 at baseline and 6 months post-treatment, but when the standard normal distribution was not satisfied, the Wilcoxon signed-rank test was used for comparative analysis.

## 3 Results

### 3.1 Baseline characteristics

This study analyzed the clinical data of 33 patients (40 eyes), including 16 men and 17 women. The mean age of the participants was 51.06 ± 11.00 years (range, 32–70 years), and the average duration of diabetes was 12.61 ± 3.91 years. All 40 eyes included in the study were diagnosed with non-proliferative diabetic retinopathy.

### 3.2 BCVA and CRT

**Figure 1:**
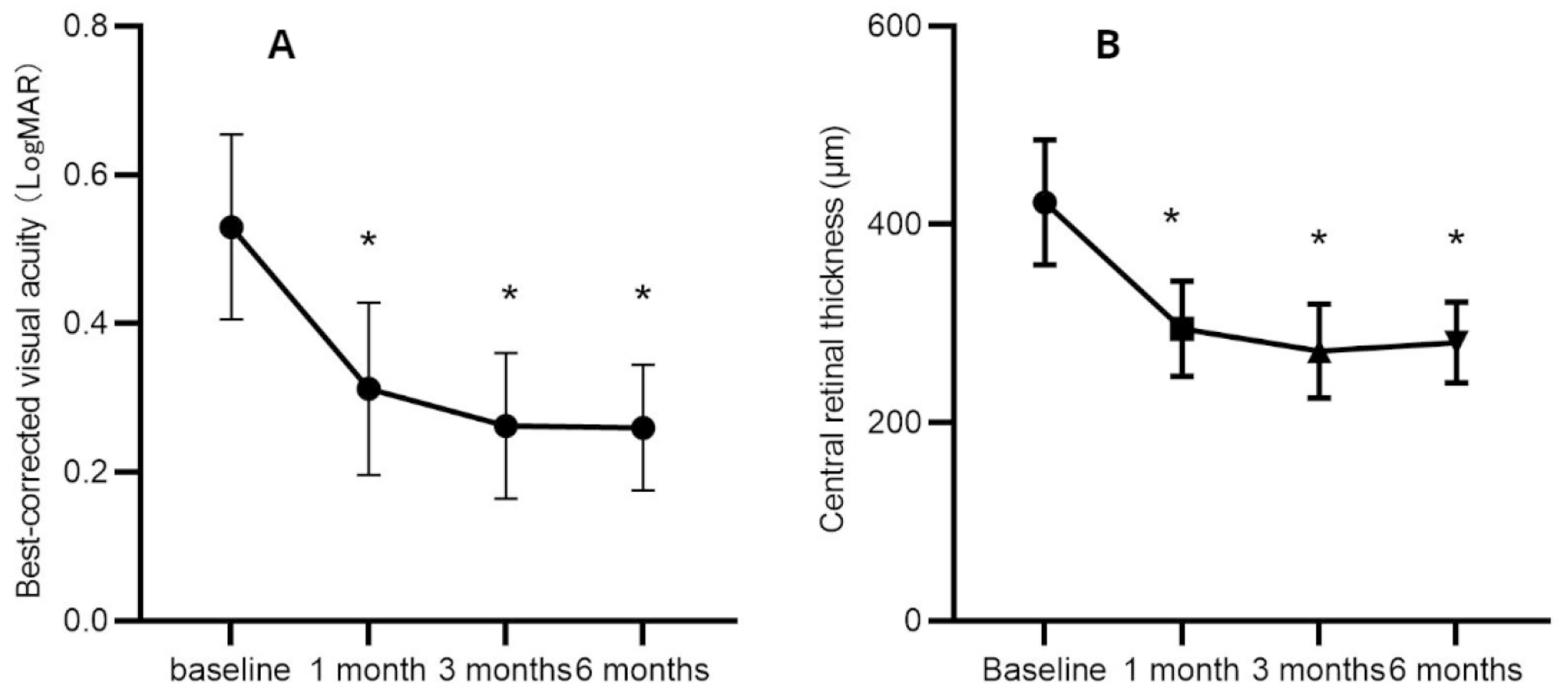
Mean changes in best-corrected visual acuity **(A)** and central retinal thickness **(B)** during the 6-month follow-up period. *p < 0.05 statistically significant difference compared with baseline values.

The BCVA and CRT measurements are summarized in Tables 1, 2, as well as illustrated in Figure 1.

**Table 1:** Comparison of BCVA and CRT at 1-, 3-, and 6-months post-treatment and baseline values.

Indices (mean ± SD)	Preoperation	1 month	3 months	6 months
BCVA (LogMAR)	0.53 ± 0.12	0.31 ± 0.12*	0.26 ± 0.10*	0.26 ± 0.08*
*p-*value	—	<0.0001	<0.0001	<0.0001
CRT (μm)	422.4 ± 63.04	294.7 ± 47.89*	272.1 ± 47.43*	281.0 ± 40.72*
*p*-value	–	<0.0001	<0.0001	<0.0001

BCVA, best-corrected visual acuity; logMAR, logarithm of minimal angle of resolution; CRT, central retinal thickness. *P < 0.05 statistically significant difference compared with baseline values.

**Table 2:** Comparison of BCVA and CRT at 1-, 3-, and 6-months post-treatment.

	BCVA (LogMAR) *p*-value	CRT *p*-value
1 vs. 3 months	0.0615	0.1939
1 vs. 6 months	0.0090	0.5621
3 vs. 6 months	0.9984	0.6013

BCVA, best-corrected visual acuity; logMAR, logarithm of minimal angle of resolution; CRT, central retinal thickness.

### 3.3 FAZ area, VD of retinal capillaries, and Ámp-P1

**Figure 2:**
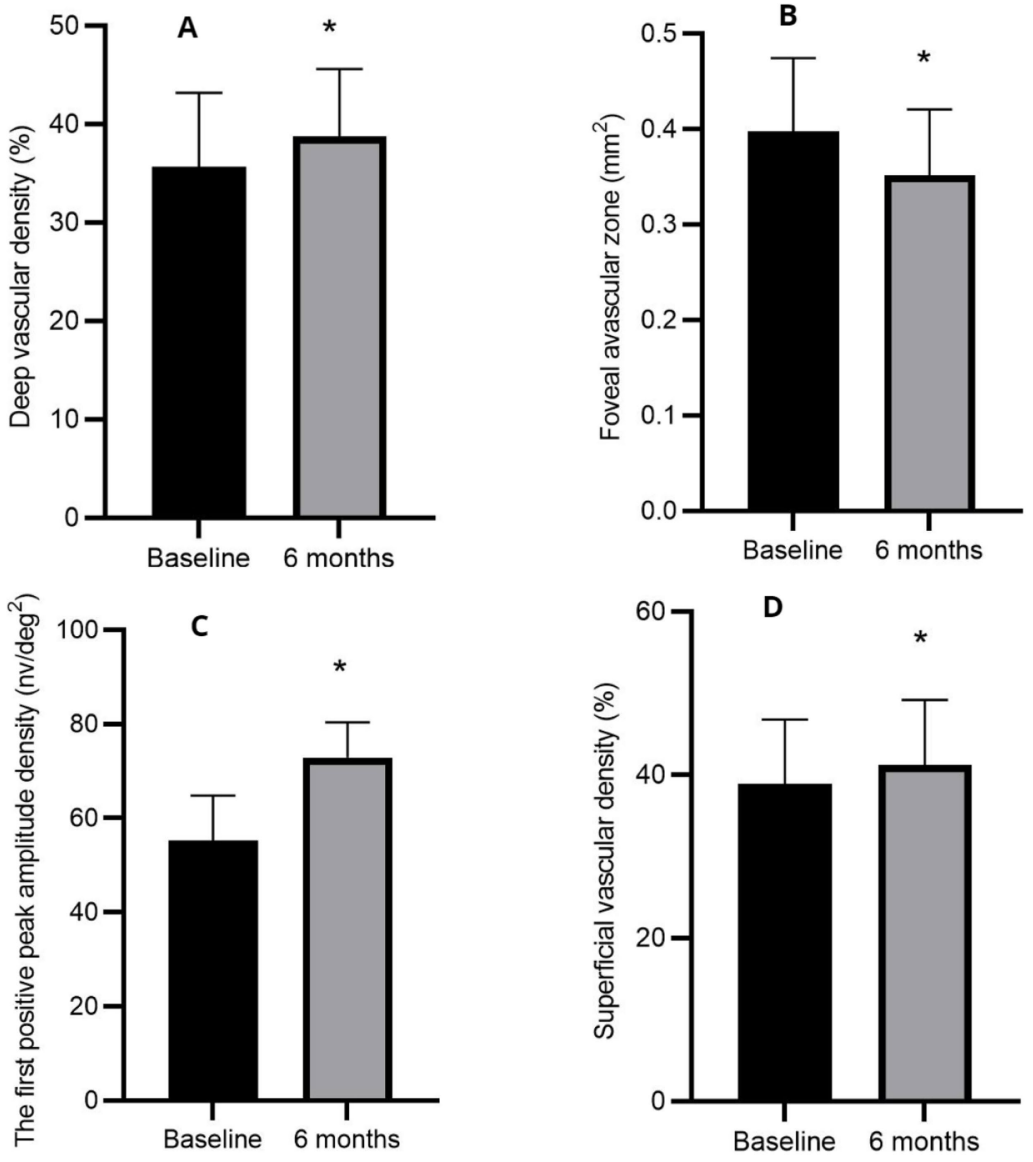
Mean changes in deep vascular density **(A)**, foveal avascular zone **(B)**, the first positive peak amplitude density **(C)**, and superficial vascular density **(D)** before and at 6 months post-treatment. *p < 0.05 statistically significant difference compared with baseline measurements.

The FAZ area, VD of retinal capillaries, and Amp-P1 are presented in Table 3 and illustrated in Figure 2.

**Table 3:** Comparative analysis of FAZ area, VD of retinal capillaries, and Amp-P1 before and 6 months post-treatment.

Indices (mean ± SD)	Preoperative	6 months	*P*
FAZ (mm^2^)	0.40 ± 0.08	0.35 ± 0.07	<0.0001
Superficial VD (%)	38.90 ± 7.88	41.21 ± 7.98	<0.0001
Deep VD (%)	35.67 ± 7.50	38.72 ± 6.90	<0.0001
Amp-P1 (nv/deg^2^)	55.30 ± 9.45	72.90 ± 7.44	<0.0001

FAZ, foveal avascular zone; VD, vascular density; Amp-P1, first positive peak amplitude density.

### 3.4 Complications

Subconjunctival hemorrhage was observed in five eyes, corneal epithelial injury occurred in two eyes, and intraocular pressure increased transiently in two eyes. No severe complications such as endophthalmitis, glaucoma, cataracts, or vitreous hemorrhage occurred in 40 eyes. Furthermore, no cardiovascular or cerebrovascular events were reported in any of the patients during the follow-up period.

### 3.5 Number of injections

The average number of injections administered to 40 eyes was 3.20 ± 0.46 over the 6-month follow-up period post-treatment.

## 4 Discussion

The prevalence of type 2 diabetes is increasing due to the acceleration of the aging process of the population and changes in lifestyle and other factors (14). Chronic hyperglycemia poses a threat to many organs of the body, especially the eyes, nervous system, kidneys, heart, and blood vessels (15). Diabetic retinopathy (DR), one of the most frequent microvascular complications of diabetes, is categorized into proliferative and non-proliferative forms. Among these, DME has the most profound impact on vision acuity in patients with non-proliferative diabetic retinopathy (16).

The treatment of DME includes retinal photocoagulation, hormones, and anti-VEGF agents. The primary treatment for DME in the past was macular laser photocoagulation, which effectively reduces macular edema, but it has a limited effect on vision acuity improvement. In recent years, anti-VEGF therapy has emerged as the preferred treatment for DME due to its demonstrated efficacy (17, 18). Within the VEGF family, VEGF-A and placental growth factor (PGF) play central roles in the pathogenesis of DME. VEGF-A promotes the proliferation and migration of endothelial cells, enhances vascular permeability, and is directly implicated in the development of macular edema. PGF exacerbates vascular leakage by mediating inflammatory responses. Aflibercept exhibits a high binding affinity for both VEGF and PGF. A meta-analysis of 43 randomized controlled trials involving 8,234 patients demonstrated that aflibercept outperformed conbercept and ranibizumab in treating DME (19). Approved by the US FDA in 2014 for DME treatment, aflibercept effectively blocks VEGF at a low concentration and has a prolonged duration of action, allowing for extended injection intervals and reducing treatment burden. These properties may explain its superior efficacy in DME management.

Despite its therapeutic advantages, the high cost of aflibercept imposes a significant economic burden on patients. The emergence of biosimilar drugs offers a promising solution to this challenge. Biosimilars are highly similar to their reference products in terms of quality, safety, and efficacy but are more cost-effective, potentially improving patient access and treatment adherence. However, the therapeutic efficacy of aflibercept biosimilars in DME is worthy of further study.

Although intravitreal anti-VEGF injections are generally considered safe, it is crucial to remain vigilant about potential ocular and systemic complications associated with the procedure. Ocular complications primarily include uveitis, endophthalmitis, retinal detachment, vitreous hemorrhage, sustained elevation of intraocular pressure, cataract progression, pain, and floaters.

Some scholars propose that the increase in VEGF concentration within the vitreous serves as a compensatory mechanism to restore macular blood perfusion. While anti-VEGF treatment effectively improves macular edema, it may also accelerate retinal capillary occlusion in patients with DME, potentially exacerbating visual impairment (20, 21). Currently, fundus fluorescein angiography remains the most effective tool for assessing the FAZ; however, it lacks the ability to differentiate between superficial and deep retinal capillary layers. In contrast, OCTA provides detailed visualization of the FAZ morphology in both the superficial and deep retinal vascular plexus. Theoretically, anti-VEGF drugs may induce the contraction of retinal arterioles, which could lead to the expansion of the FAZ, particularly in patients with pre-existing macular ischemia (22). In recent years, mfERG has emerged as a non-invasive, objective method for assessing visual function, proving valuable in evaluating retinal vascular diseases such as diabetes retinopathy and guiding treatment strategies (23, 24).

In this study, we evaluated changes in BCVA, CRT, FAZ area, VD of retinal capillaries, and Amp-P1 before and after treating DME with an aflibercept biosimilar. We also assessed the efficacy and safety of the aflibercept biosimilar in managing DME. The results demonstrated a significant improvement in BCVA at 1-, 3-, and 6-months post-treatment compared to baseline, alongside a significant reduction in CRT and macular edema. These findings align with previous studies, which reported comparable efficacy between the aflibercept biosimilar (QL1207) and the reference aflibercept in treating neovascular age-related macular degeneration (25). However, unlike previous research, this study uniquely explores the effects of the aflibercept biosimilar (QL1207) on macular perfusion and functional outcomes in DME patients before and after treatment.

The Amp-P1 was significantly higher at 6 months post-treatment compared to baseline (*p* < 0.05), suggesting improved macular function following treatment. Additionally, the study revealed a reduction in the FAZ area and an increase in VD after treatment, indicating enhanced macular perfusion after anti-VEGF therapy. These findings are consistent with previous research on the effects of conbercept on macular perfusion in patients with DME (26). Notably, no severe complications such as endophthalmitis, glaucoma, cataract progression, or vitreous hemorrhage were observed in any of the 40 treated eyes. Furthermore, none of the patients experienced cardiovascular or cerebrovascular events during the treatment period.

Intravitreal injections of the aflibercept biosimilar significantly reduce macular edema in patients with DME, improving their BCVA, macular perfusion status, and macular function. However, this study has several limitations, including its retrospective design, small sample size, and relatively short follow-up period. The long-term efficacy of the aflibercept biosimilar in treating DME remains to be confirmed through large-scale, prospective studies with extended follow-up. Additionally, future research should compare the therapeutic effects of the aflibercept biosimilar (QL1207) with other anti-VEGF agents to further elucidate its relative efficacy and safety.

## Data Availability

The original contributions presented in the study are included in the article/supplementary material, further inquiries can be directed to the corresponding author.
