# Corrigendum: Efficacy and safety of intravitreal injection of aflibercept biosimilar for treating diabetic macular edema

**DOI:** 10.3389/fmed.2025.1620932

**Published:** 2025-05-19

**Authors:** Gaixia Zhai, Chao Sun, Xia Zhang, Yuanzhen Su

**Affiliations:** Zibo Central Hospital, Zibo, China

**Keywords:** aflibercept biosimilar, diabetic macular edema, foveal avascular zone, vascular density, multifocal electroretinography

In the published article, the reference for (5) was incorrectly written as “Georgalas, L, Tservakis, I, Kiskira, EE, Petrou, P, Papaconstantinou, D, and Kanakis, M. Efficacy and safety of intravitreal dexamethasone implant in treatment-resistant diabetic macular edema: six-month results. *Cesk Slov Oftalmol*. (2025) 81:330–7. doi: 10.31348/2025/4.” It should be “Berhuni M, Yılmaz İE, Gizem GS, Özcan ZÖ, Doğan L. Efficacy and safety of intravitreal dexamethasone implant in treatment-resistant diabetic macular edema: six-month results. *Cesk Slov Oftalmol*. (2025) 81:1–6. doi: 10.31348/2025/4.”

The authors apologize for this error and state that this does not change the scientific conclusions of the article in any way. The original article has been updated.
